# Prediction of unplanned cesarean section using measurable maternal and fetal characteristics, Ethiopia, a retrospective cohort study

**DOI:** 10.1186/s12884-024-06308-2

**Published:** 2024-02-23

**Authors:** Bezawit Melak Fente, Mengstu Melkamu Asaye, Temesgen Worku Gudayu, Muhabaw Shumye Mihret, Getayeneh Antehunegn Tesema

**Affiliations:** 1 Department of General Midwifery, School of Midwifery, College of Medicine & Health Sciences, University of Gondar, Gondar, Ethiopia; 2 Department of Women’s and Family Health, School of Midwifery, College of Medicine & Health Sciences, University of Gondar, Gondar, Ethiopia; 3 Department of Clinical Midwifery, School of Midwifery, College of Medicine & Health Sciences, University of Gondar, Gondar, Ethiopia; 4 Department of Epidemiology and Biostatistics, Institute of Public Health, College of Medicine and Health Sciences, University of Gondar, Gondar, Ethiopia

**Keywords:** Unplanned cesarean section, Prediction model, Ethiopia

## Abstract

**Background:**

When a pregnant woman experiences unusual circumstances during a vaginal delivery, an unplanned cesarean section may be necessary to save her life. It requires knowledge and quick assessment of the risky situation to decide to perform an unplanned cesarean section, which only occurs in specific obstetric situations. This study aimed to develop and validate a risk prediction model for unplanned cesarean sections among laboring women in Ethiopia.

**Method:**

A retrospective follow-up study was conducted. The data were extracted using a structured checklist. Analysis was done using STATA version 14 and R version 4.2.2 software. Logistic regression was fitted to determine predictors of unplanned cesarean sections. Significant variables were then used to develop a risk prediction model. Performance was assessed using Area Under the Receiver Operating Curve (AUROC) and calibration plot. Internal validation was performed using the bootstrap technique. The clinical benefit of the model was assessed using decision curve analysis.

**Result:**

A total of 1,000 laboring women participated in this study; 28.5% were delivered by unplanned cesarean section. Parity, amniotic fluid status, gestational age, prolonged labor, the onset of labor, amount of amniotic fluid, previous mode of delivery, and abruption remained in the reduced multivariable logistic regression and were used to develop a prediction risk score with a total score of 9. The AUROC was 0.82. The optimal cut-off point for risk categorization as low and high was 6, with a sensitivity (85.2%), specificity (90.1%), and accuracy (73.9%). After internal validation, the optimism coefficient was 0.0089. The model was found to have clinical benefits.

**Conclusion:**

To objectively measure the risk of an unplanned Caesarean section, a risk score model based on measurable maternal and fetal attributes has been developed. The score is simple, easy to use, and repeatable in clinical practice.

## Introduction

Cesarean section (CS) delivery is the delivery of the fetus, placenta, and membranes by an abdominal and uterine wall incision at or after 28 weeks of pregnancy [1]. If pregnant women face unusual conditions during a vaginal delivery, unplanned CS can be a life-saving operation [2].

World Health Organization (WHO) recommends the ideal rate of CS to be between 5 and 15%. Despite this recommendation, the CS rate is rapidly increasing; even the causes for the continuous increase in CS rates are not fully understood [3, 4]. Each year, 20 million CS are performed globally [3], with rates ranging from 7.1% in Sub-Saharan Africa to 63.4% in Eastern Asia [5]. In Ethiopia CS the prevalence was 39.1% [6]. On the contrary, there is research that indicates that, over a certain period, rising unplanned CS rates may be related to higher rates of maternal and neonatal morbidity [7, 8].

Unplanned CS, is associated with increased maternal morbidity and mortality, compared to a scheduled CS [9]. Severe hemorrhage, complications with the quick administration of general anesthesia, and inadvertent damage to the mother and child are among the morbidity linked with unplanned CS [8, 10]. Intrapartum variables such as labor dystocia, fetal distress, and umbilical cord prolapse are reasons for unplanned CS [1]. The most common predictors of unplanned CS were parity, residency, antepartum hemorrhage, prior cesarean section, number of visits Antenatal care(ANC), induction of labor, hypertensive disorder, and induction of labor [4, 6, 10–15]

A trial of vaginal labor should be done for expecting women who show no clear indicators of CS. It is also critical to monitor changes in maternal and fetal health status in order to protect mothers and babies and prepare for unplanned CS. It is only performed in specific obstetric situations and requires knowledge and a timely assessment of the dangerous situation for unplanned CS as soon as possible. Identifying an urgent scenario that could be potentially life-threatening is one of the most difficult challenges in obstetrics. Risk prediction of obstetric conditions demanding unplanned CS could be very helpful for both clients' and healthcare providers’ preparedness and timely responses. It helps clinicians, in decision-making with the hope of decreasing maternal and neonatal morbidity and mortality and lowering personal and institutional healthcare costs. It might be beneficial, especially in areas with limited resources. Despite this huge problem, few studies indicate unplanned CS in Ethiopia. Previous studies have focused on determining the associated factors that contribute to unplanned CS. A predictive model enables real-time mothers’ unplanned CS risk stratification, which guides primary attention to care for maternal and neonatal health good health outcomes. There are no studies on prediction models related to unplanned CS based on clinical and demographic variables in Ethiopia. Therefore, this study aims to develop and validate of risk prediction model to predict unplanned CS using easily available variables.

## Method and materials

### Study design, setting, and population

A retrospective follow-up study was conducted at Debre Markos Comprehensive and Specialized Hospital (DMCSH) from January 1, 2020, to May 30, 2022, and data were extracted (collected) from September 1, 2022, to October 2022. Debre Markos Comprehensive and Specialized Hospital is located in Debre Markos Town, which is 299 km from Addis Ababa, Ethiopia's capital city, and 265 km from Bahir-Dar, the capital city of Amhara Regional State. The hospital is one of the largest tertiary-level referral facilities in the Amhara region. (1) All laboring women who give birth from January 1, 2020, to May 30, 2022; (2) A vaginal examination was done before birth, (3) There was no sign of fetal distress. While, multiple pregnancies, breech and transverse presentation, severe fetal malformation-related problems, failure to deliver vaginally in an outside hospital and transfer to our hospital, and direct CS with specific indications were excluded.

### Sample size and sampling technique

Sample size calculation using a Rule of thumb of at least 10 events per predictor was used [16]. In the prediction model, each predictor needs at least ten events (dependent variable) we had a total of fourteen predictors (independent variables). To get the estimated sample size, we have used the previous incidence of unplanned CS (13.7%). Therefore, fourteen predictors are multiplied by ten events (rule of thumb) divided by the incidence. Finally, the calculated sample size was 1,021 participants. After all, 1000 participants were included in this study, and 21 were missed data. We have used a previous study incidence of unplanned CS is 13.7% [17].

$${\text{N}}=\left({{\text{n}}}^{*}10\right)/{\text{I}},$$

$$({14}^{*}{10}^{*}100)/13.7=\mathrm{1,021}.$$

A simple random sampling technique (method) was employed to select participants using the medical registration number of a delivered mother from the delivery registration book.

### Study variables and measurements

#### Dependent variable

The dependent variable for this study was the mode of delivery. Normal vaginal delivery (NVD) and unplanned CS. Unplanned CS was defined as a case in which the procedure of vaginal delivery was discontinued due to the occurrence of fetal distress or other reasons [1].

#### Independent variables

The independent variable demographic characteristics of delivered women (Maternal age, weight, and residence), pregnancy history (Gravidity, parity, previous mode of delivery, number of ANC), perinatal complications (amniotic fluid, hypertensive disorders of pregnancy, placental abruption, anemia (hemoglobin < 11 g/dl), PROM, comorbidity(chronic hypertension, DM, heart diseases, etc.), neonatal characteristics(Birth weight, gestational age), and Labor process( Onset of labor(labor starting by inducing or spontaneous), prolonged labor(labor was longer than 24 h)).

### Data collection and quality assurance

A data extraction checklist was prepared on the Kobo Toolbox web-based tool for the collection of data from the mother's medical records. The checklist was arranged into demographic characteristics, pregnancy history, perinatal complications, neonatal features, and labor process. Data collectors were trained for two days by the principal investigator. Frequent and timely supervision of data collectors was undertaken.

### Statistical analysis

Data were collected using the Kobo Toolbox. Collected data were exported to STATA version 14 and R Software version 4.2.2 for data management and analysis. Missing data were handled by multiple imputations missing handling technique by assuming missing at random. Multi-collinearity among independent predictors was checked by the Variance Inflation Factor (VIF).

Transparent Reporting of a Multivariable Prediction Model for Individual Prognosis Or Diagnosis (TRIPOD) guideline used for developing and reporting prediction model [18]. Tables and figures were used to describe the characteristics of the study participants.

Logistic regression analysis was used to evaluate which variables are the most powerful to predict unplanned CS [19]. A bivariable regression analysis was used to obtain insight into the association of each potential determinant of unplanned CS and for inclusion in multivariable regression analysis. Variables with *p*-value < 0.25 in the bivariable analysis were fitted to the multivariable regression analysis. After a stepwise backward elimination technique was used, the role of each predictor in the multivariable analysis was assessed by the likelihood ratio test. The unplanned CS prediction model was developed from significant variables in the reduced multivariable regression model. Regression coefficients of the reduced model were used as a measure of the effect of predictor variables on the probability of unplanned CS.

A risk score was developed using identified coefficients for which the weights were defined as the quotient of the corresponding estimated coefficient from a reduced multivariable regression analysis divided by the smallest beta-coefficient and rounded to the nearest integer. We determined the total score for each individual by assigning the points for each variable present and adding them up. The score was transformed to a dichotomous; allowing each pregnant woman to be classified as a high or low risk of unplanned CS. The model performance was assessed using discrimination power and calibration. The area under the receiver operating characteristic curve was used to evaluate the discrimination power of the developed prediction model. Model calibration was assessed using a calibration plot and *p*-value to ensure the reliability of the prediction models. To choose the optimal cut-off point to separate the risk score of the patients as high and low, the Youden index method was used [20]. For evaluation of the predictive efficiency of the optimal cut-off values of the models, sensitivities, specificities, PPV (Positive Predictive Value), NPV (Negative Predictive Value), likelihood ratio, and accuracy were used. Internal validation was performed using the bootstrap procedure replicating the sample 1,000 times to estimate how the performance of the prediction model developed on the development set would be on a hypothetical set of new patients. Decision curve analysis was used to assess the clinical benefit of the prediction model.

## Results

### Demographic characteristics of delivered women

A total of 1,000 laboring women were included in this study with a response rate of 97.9%. The median age of mothers was 27 with an Interquartile Range (IQR) of 24 to 30 years and the majority of them were aged between 20–34 years. More than three-fourths of the mothers were urban residents from them 23.4% of mothers had delivered by unplanned CS and almost all of the mothers were married (Table 1).

**Table 1:** Demographic characteristics of delivered women

Variable	Category	Mode of delivery	
		**NVD**	**Unplanned CS**
Age	< 20	31(1.3)	18(1.8)
	20–34	611(61.1)	236(23.6)
	> = 35	73(7.3)	31(3.1)
Residence	Urban	628(62.8)	234(23.4)
	Rural	87(8.7)	51(5.1)
Marital status	Married	699(69.9)	273(27.3)
	Single	9(0.9%)	11(1.1%)
	Divorce/Widow	7(0.7%)	1(0.1%)
Weight of mother	≥ 50	654(65.4%)	262(26.2%)
	< 50	48(4.8%)	19(1.9%)
MUAC	> = 24	580(58.0%)	232(23.2%)
	< 24 cm	121(12.1%)	49(4.9%)

^*^NVD Normal Vaginal Delivery, *CS Cesarean Section

### Perinatal and intrapartum characteristics

Nearly two-thirds (59.8%) of the study participants were multigravida. More than half (54.6%) of the study subjects were multipara, and the majority (76.56%) of the 546 multiparous women gave birth vaginaly. Concerning the previous mode of delivery, about 71 of the study participants gave birth via CS and a considerable proportion (46.48%) of them delivered vaginally despite previous CS scar. Seventy-one (7.8%) of the participants underwent induction of labor for the indexed childbirth (Table 2).

**Table 2:** Perinatal and intrapartum characteristics

Variable	Category	Mode of delivery	
		**NVD(n,%)**	**Unplanned CS (n,%)**
Gravidity	Multigravida	454(45.4%)	144(14.4%)
	Primigravida	261(26.1%)	141(14.1%)
Parity	Multiparous	418 (41.8%)	128(12.8%)
	Nulliparous	297(29.7%)	157(15.7%)
Mode of previous delivery	SVD	682(68.2%)	247(24.7%)
	CS	33(3.3%)	38(3.8%)
PROM	Yes	18(1.8%)	23(2.3%)
	No	683(68.3%)	268(26.8%)
	Yes	32(3.2%)	17(1.7%)
Comorbidity	No	703(70.3%)	275(27.5%)
	Yes	12(1.2%)	10(1.0%)
Anemia	No	684(68.4%)	272(27.2%)
	Yes	31(3.1%)	13(1.3%)
Amount of AF	Normal	687(68.7%)	252(25.2%)
	Abnormal	28(2.8%)	33(3.3%)
Onset of labor	Spontaneous	682(68.2%)	247(24.7%)
	Induced	33(3.3%)	38(3.8%)
Prolonged labor	No	680(68.0%)	239(23.9%)
	Yes	35(3.5%)	46(4.6%)
AF status	Clear	669(66.9%)	206(20.6%)
	Abnormal	46(4.6%)	79(7.9%)
Gestational age	< 40	500(50.0%)	166(16.6%)
	> = 40	215(21.5%)	119(11.9%)
Birth Weight	< 2500	89(8.9%)	33(3.3%)
	2500–4000	618(61.8%)	246(24.6%)
	> = 4000	8(0.8%)	6(0.6%)

^*^AF Amniotic Fluid, *NVD Normal Vaginal Delivery, *CS Cesarean Section

### Development of prediction model for unplanned CS

#### Predictor selection for prediction of unplanned CS

From routinely collected data on demographic, perinatal, and intrapartum characteristics of laboring women, 20 potential predictors were considered for further evaluation to be included in the prediction model. 17 predictors were identified using univariate regression analysis. The variables including age, weight, MUAC, hemoglobin, PIH, PROM, birth weight, and number of ANC were excluded for insignificant association. Those predictors with *P* < 0.25 were incorporated in the multivariate logistic regression analysis. By using backward stepwise selection, predictors with *P* < 0.15 with likelihood ratio test for their role on the regression model were finally seven predictors left in the reduced model and selected as candidates for prediction model development; including Parity, amount of AF, Gestational age, Prolonged labor, onset of labor, Amniotic fluid status, previous mode of delivery, and Abruption(yes) (Table 3).

**Table 3:** Univariate and multivariate analyses of factors associated with Unplanned CS

variable	Category	Univariate		Multivariate	
		**Coef. (95%CI)**	***p*-value**	**Coef. (95%CI)**	***p*-value**
Age	< 20	0.407(-0.19–1.00)	0.183	NA	
	20–34	Ref			
	> = 35	0.094(-0.35–0.54)	0.677		
Residence	Urban	Ref			
	Rural	0.461(0.085–0.836)	0.016	0.342(-0.11–0.79)	0.141
Marital status	Married	Ref			
	Single	1.116(0.224–2.007)	0.014	-1.11(-2.12–0.115)	0.069
	Divorce/Widow	-1.031(-3.131–1.069)	0.336	-2.04(-4.67–0.58)	0.128
Weight of mother	Normal				
	Underweight	0.039(-0.503–0.581)	0.888	NA	
MUAC	> = 24	Ref			
	< 24 cm	0.051(-0.31–0.412)	0.782	NA	
Anemia	No	Ref			
	Yes	0.14(-0.51–0.789)	0.674	NA	
Gravidity	Multigravida	Ref		Ref	
	Primigravida	0.39(0.114–0.667)	0.006	0.265(-0.44–0.975)	0.464
Parity	Multiparous	Ref		Ref	
	Nulliparous	0.344(0.069–0.618)	0.014	0.762(0.025–1.498)	**0.043**
Comorbidity	No	Ref		Ref	
	Yes	0.731(-0.119–1.58)	0.092	0.507(-0.516–1.531)	0.331
Mode of previous delivery	SVD	Ref		Ref	
	CS	1.75(1.353–2.151)	0.000	2.824(2.327–3.321)	**0.000**
PIH	No	Ref		NA	
	Yes	0.097(-0.572–0.766)	0.776		
Abruption	No	Ref		Ref	
	Yes	1.197(0.565–1.83)	0.000	1.277(0.516–2.038)	**0.001**
PROM	No	Ref		NA	
	Yes	0.277(-0.328–0.882)	0.369		
Amount of AF	Normal	Ref		Ref	
	Abnormal	1.14(0.617–1.664)	0.000	0.746(0.093–1.4)	**0.025**
Onset of labor	Spontaneous	Ref		Ref	
	Induced	1.25(0.763–1.745)	0.000	0.963(0.366–1.56)	**0.002**
Prolonged labor	No	Ref		Ref	
	Yes	1.40(0.937–1.87)	0.000	1.176(0.618–1.733)	**0.000**
AF status	clear	Ref		Ref	
	Abnormal	1.81(1.41–2.21)	0.000	1.93(1.48–2.396)	**0.000**
Gestational age	< 40	Ref		Ref	
	> = 40	0.534(0.250–0.817)	0.000	0.472(0.118–0.826)	**0.009**
Birth Weight	< 2500	Ref		NA	
	2500–4000	0.070(-0.35- 0.496)	0.744		
	> = 4000	0.704(-0.42- 1.835)	0.222		
Number of ANC	> = 4	Ref		NA	
	< 4	0.177(-0.45–0.099)	0.290		

^*^MUAC Middle Upper Arm Circumference, *PIH* Pregnancy Induce Hypertension, *PROM* Premature Rupture of Membrane, *AF* Amniotic Fluid, *ANC* Antenatal Care, ^*^NA Not applicable

#### Prediction Model development for unplanned CS

The theoretical design of this study found to be

$$\mathrm{Unplanned}\;\mathrm{CS}(t_0+1)=\mathrm f\;(\mathrm{Parity}(t_0)+\mathrm{AF}\;(t_0)+458\;\mathrm{Gestional}\;\mathrm{age}\;(t_0)+\mathrm{Prolonged}\;\mathrm{labor}(t_0)+\mathrm{Amnioticfluid}\;(t_0)+\mathrm{Onset}\;\mathrm{of}\;\mathrm{labor}\;(t_0)+\mathrm{Previous}\;\mathrm{mode}\;\mathrm{of}\;\mathrm{delivery}\;(t_0)+\mathrm{Abruption}\;(t_0))$$

Based on the regression coefficients of predictors in the reduced model, the linear predictor model for estimated probability unplanned CS of laboring women could be calculated as:

Linear prediction model of unplanned CS

$$=-2.37+1.022\;\mathrm{Parity}\;(\text{Nullparious})+0.698\;\mathrm{AF}\;(\text{Abnormal})+0.458\;\mathrm{Gestational}\;\mathrm{age}\;(>=40)+1.18\;\mathrm{Prolonged}\;\mathrm{labor}\;(\text{yes})+1.03\;\mathrm{onset}\;\mathrm{of}\;\mathrm{labor}(\text{induced})+1.94\;\mathrm{Aminoic}\;\mathrm{fluid}\;(\text{abnormal})+2.79\;\mathrm{Previous}\;\mathrm{mode}\;\mathrm{of}\;\mathrm{delivery}\;(\text{cs})+1.34\;\mathrm{Aburaption}\;(\text{yes})$$

The equation provided above estimates the probability of unplanned CS based on the status they have on the predictors. Laboring women had 2 possible values in each predictor. If the risk presents for the predictors corresponding beta coefficients will be added otherwise will be taken as zero if the risk is absent.

**Fig. 1:**
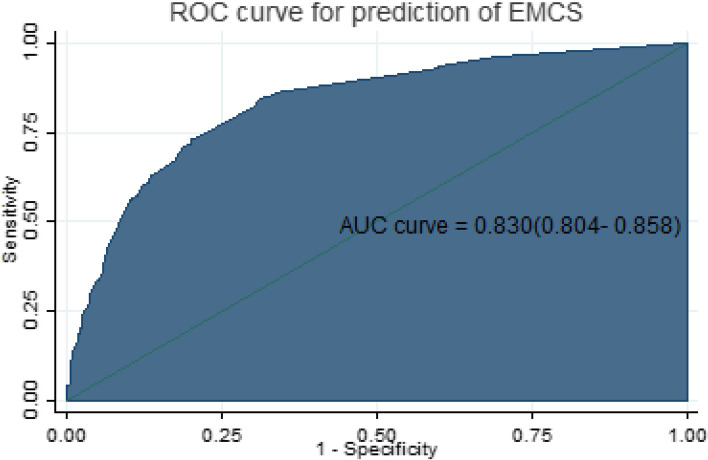
Area under the ROC curve for the prediction of EmCS

The AUC of the reduced model was 0.830(95% CI: 0.804–0.858) (Fig. 1). As the calibration plots and test; a *P*-value of 0.076, the estimated probability of unplanned CS agreed very well with the observed probability which indicates the model does not misrepresent the data.

Validation of the model using the bootstrap technique revealed little evidence of undue influence by particular observations, with an optimism coefficient of 0.00087 and an AUC of 0.80 (adjusted 95% CI: 0.77–0.84).

### Risk score development

For simplicity of clinical use and to avoid sophisticated risk calculation, a Simplified risk score predictor model for the estimated risk of unplanned CS was developed by rounding the regression coefficients to the nearest integer after weighting with the least coefficient.

The estimated risk score of unplanned CS was calculated as

$$\mathrm{Simplified}\;\mathrm{risk}\;\mathrm{score}=2^{\ast\;}\mathrm{Parity}\left(\text{Nulliparous}\right)+2^{\ast\;}\mathrm{AF}\left(\text{Abnormal}\right)+1^\ast\;\mathrm{gestational}\;\mathrm{age}\left(>=40\right)+3^{\ast\;}\mathrm{Prolongedlabor}\left(\text{yes}\right)+2^{\ast\;}\mathrm{onset}\;\mathrm{of}\;\mathrm{labor}\left(\text{induced}\right)+3^\ast\mathrm{Amniotic}\;\mathrm{fluid}\;\mathrm{abnormal})+3^{\ast\;}\mathrm{Previous}\;\mathrm{mode}\;\mathrm{of}\;\mathrm{delivery}\left(\text{cs}\right)+2^\ast\text{Abruption}(\text{yes})$$

Developed prediction models using original beta and simplified risk score had similar discrimination ability (AUC of simplified risk score was 0.820(95% CI: 0.790–0.849)) as well as comparable sensitivity and specificity for their optimal cut-off points. The possible minimum and maximum scores for laboring women can be 0 and 18, respectively. The optimal cutoff point was suggested by the Youden index [20] to dichotomize the risk of unplanned CS as high risk and low risk found to be 6. Patients with a score less than 6 were low risk and those with 6 and above were at high risk of unplanned CS.

Table 4 shows the predictive efficiency of different possible cut-off points of the simplified risk score. The cut-off points 6 had a sensitivity of 85.2%, specificity of 90.1%, Positive predictive value of 66.1%, Negative predictive value of 75.4%, and Accuracy of 73.9%.

**Table 4:** Performance of risk score at different cutoff points for Unplanned CS

cut-off points	Sensitivity	specificity	accuracy	NPV	PPV	LR +	LR-
2	98.63	58.46	62.5	84.27419	41.07	1.41	0.56
4	92.31	83.77	73.1	79.65	53.22	2.95	0.63
6	**85.15**	**90.12**	**73.9**	**75.44**	**66.11**	**4.08**	**0.72**
8	82.28	91.04	73.6	73.71	73.42	4.93	0.82
10	80.56	94.58	78.5	72.35	80.25	9.47	0.89
12	72.10	95.86	82.0	71.90	85.71	15.05	0.96

^*^PPV (Positive Predictive Value), *NPV (Negative Predictive Value),

### Risk classification

According to the developed risk score, of all the laboring women included in the study, 618 were categorized under the low-risk group and the proportion of unplanned CS was 10.5%. 384 laboring women were found to be high risk with a percentage of unplanned cesarean delivery of 18.0% (Table 5).

**Table 5:** Risk classification for unplanned cesarean section based on simplified risk score

**Risk group**	Prediction Model Based on Maternal Characteristics		
	Number of women	Mode of delivery	
		NVD	Unplanned CS
**low risk**	618(61.8%)	513(51.3%)	105(10.5%)
**High risk**	382(38.2%)	202(20.2%)	180(18.0%)
Total	1,000(100%)	715(71.5%)	285(28.5%)

^*^NVD Normal Vaginal Delivery, *CS Cesarean Section

### Decision curve analysis

**Fig. 2:**
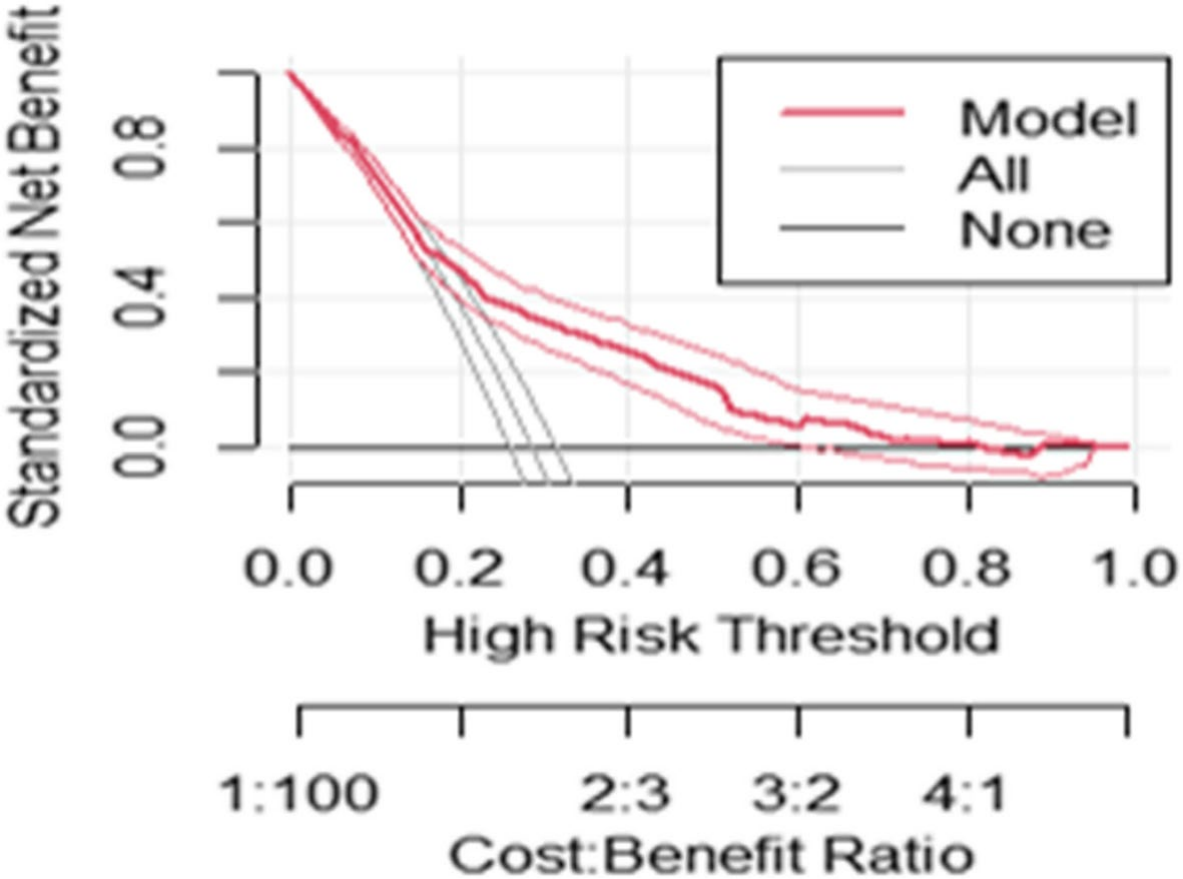
Decision curve plotting net benefit of the model against threshold probability and corresponding cost–benefit ratio

The aim of developing this prediction model is for early differentiation of those who will be delivered by unplanned CS so that they will be given critical attention to receive timely and appropriate care. As Fig. 2 shows, the model has the highest net benefit across the entire range of threshold probabilities, which indicates that the model has the highest clinical and public health value. Hence, surgical decision made using the model has a higher net benefit than not doing for all or doing for all regardless of their risk threshold (Fig. 2).

## Discussion

The present study was designed to develop and validate risk scores to predict unplanned cesarean section using maternal and fetal characteristics among laboring women who gave birth. Thus, predicting the probability of unplanned CS in laboring women is critical in order to take appropriate action. The unplanned CS scores could be used in combination with other clinical information to assist laboring mothers with guidance, expectations, and decision-making.

This study developed a novel risk score in the country for categorizing laboring women's risk of unplanned CS at the time of admission to labor. Demographic and clinical characteristics of laboring women were considered to determine predictors of risk of unplanned CS and to develop a risk prediction model. After the assessment of the association between predictors and unplanned CS, nulliparous, AF status, gestational age, prolonged labor, onset of labor, amount of amniotic fluid, previous mode of delivery, and abruption were selected as predictors.

This score is built up with predictors, widely available in current practice, simple to investigate, and quite affordable. Some of these parameters had already proven to be predictors of unplanned CS in previously published studies. Nulliparous was among the set of predictors studies done in China [2], Columbia [10], United Kingdom [21], Germany [22], Palestinian [15], Tanzania [23]. oligohydraous AF amount (Abnormal) has become part of a risk factor for unplanned CS evidenced by studies done in China [2] and Palestinian [15]. This is in agreement with the results of research and review articles [2, 14, 22, 24]. Prolonged labor China [25], wollo [11], Addis Ababa 2019(33), Palestinian (52). The onset of labor (induced) China [2], United Kingdom [21], United States [26], Addis Ababa 2019(33), and Palestinian [15], Amniotic fluid (abnormal) China [2], United Kingdom [21]. Previous cesarean delivery was a significant factor in predicting unplanned CS. This finding is consistent with the results of other studies in China [2], Germany [22], wollo [11], Addis Ababa [14], Addis Ababa 2019 [24], Palestinian [15], and Debre Tabor [6]. Abruption is also a predictor of PTB in line with other studies conducted in China [2], Nigeria [27], the United Kingdom [21], and wollo [11].

Combining the above predictors the developed risk score results AUC of 0.82 which is a good accuracy [28]. The prediction accuracy to differentiate laboring women's risk of unplanned CS is 82% which is determined by the status of each predictor. This discrimination ability of our model was higher compared to the AUC of the prediction model in China [2] with AUC of 0.787, the Netherlands with an AUROC of 0.58 [29], and another study with AUC of 0.74 [10]. The difference in discrimination ability might be because the scores were developed with different combinations of demographic and clinical parameters.

In ordinary clinical and public health practice, the simplified risk score generated using the regression models is more convenient to use than the regression models and has equivalent discrimination. Without any advanced laboratory or imaging testing, this study measured the predicted performance of a model based on maternal features during pregnancy and labor. Furthermore, By using the Yoden index as an ideal cut point, we found that this prediction model's sensitivity, specificity, PPV, NPV, and accuracy achieved 85.15%, 90.12%, 75.44%, 66.11%, and 73.9%, respectively, at a score cutoff of 6.

The developed model was well calibrated which shows its reliability and showed similar discrimination performance after internal validation which indicates it is capable of predicting unplanned cesarean in independent sets of women with similar accuracy. The decision curve analysis revealed the developed risk score for early categorization of risk-laboring women for unplanned cesarean section has better clinical benefit compared to doing for none and doing for all laboring women in a wide range of threshold probabilities. Therefore, priority focus should be given based on the prediction model that had a greater cost–benefit ratio than doing for none and doing for all laboring women regardless of the prediction probability.

### Strength and limitations

The strengths of the study were, first that it was conducted with an adequate number of participant outcomes for predicting unplanned CS, which helped construct the model using a sufficient number of predictor variables and protect overfitting. Second, we have developed a simple risk score for unplanned CS which enables clinicians and patients to make personalized predictions easily and quickly. In addition, our prediction tool can serve as a guide for local governments and health departments to improve labor and delivery outcomes. The study was not without limitations, it would have been better if it had been conducted using a prospective follow-up study design. In retrospectively collected data, some variables for the prediction of unplanned CS might have been missed. However, a risk score developed using retrospectively collected data is still important in resource-limited settings like Ethiopia. Besides, the model was not externally validated using an independent dataset. It would have been better if it had gone through external validation to ensure its prediction capability when applied to other contexts.

## Conclusions and recommendations

The developed prediction model includes nulliparous, AF status, gestational age, prolonged labor, the onset of labor, amount of amniotic fluid, previous mode of delivery, and abruption as predictors of Unplanned CS. The risk score was able to predict unplanned CS of laboring women with good discrimination ability and clinical benefit. Hence, this prediction score offers an opportunity to give appropriate and timely intervention which therefore improves maternal and fetal outcomes. We recommended that clinicians use it may assist in clinical decision-making and that researchers validate the prediction tool in another context.

## Data Availability

The data sets used and analyzed during the current study were available from the corresponding author upon reasonable request.
